# Impacts of the *SOAT1* genetic variants and protein expression on HBV-related hepatocellular carcinoma

**DOI:** 10.1186/s12885-021-08245-1

**Published:** 2021-05-26

**Authors:** Yulong Chen, Xunjun Yang, Yao Chen, Guorong Chen, Cheryl A. Winkler, Ping An, Jianxin Lyu

**Affiliations:** 1 Key Laboratory of Laboratory Medicine, Ministry of Education, Zhejiang Provincial Key Laboratory of Medical Genetics, Wenzhou Medical University, Wenzhou, Zhejiang China; 2 Department of Laboratory Medicine, The Second Affiliated Hospital & Yuying Children’s Hospital of Wenzhou Medical University, Wenzhou, Zhejiang China; 3 Department of Pathology, The First Affiliated Hospital of Wenzhou Medical University, Wenzhou, Zhejiang China; 4 Basic Science Program, Frederick National Laboratory for Cancer Research, Frederick, MD USA; 5 Zhejiang Provincial People’s Hospital, Affiliated Hospital of Hangzhou Medical College, Zhejiang Hangzhou, China

**Keywords:** Hepatocellular carcinoma, SOAT1, Single nucleotide polymorphism, Susceptibility

## Abstract

**Background:**

Hepatitis B virus (HBV)-related hepatocellular carcinoma (HCC) remains a major public health problem and its pathogenesis remains unresolved. A recent proteomics study discovered a lipid enzyme Sterol O-acyltransferase (SOAT1) involvement in the progression of HCC. We aimed to explore the association between *SOAT1* genetic variation and HCC.

**Methods:**

We genotyped three exonic *SOAT1* variants (rs10753191, V323V; rs3753526, L475L; rs13306731, Q526R) tagging most variations in the gene, in 221 HCC patients and 229 healthy individuals, to assess the impact of *SOAT1* gene variation on risk of HCC occurrence. We further conducted immunohistochemistry to compare SOAT1 protein expression levels in 42 paired tumor and adjacent non-tumor tissues.

**Results:**

We found that rs10753191 (Odds ratio (OR) = 0.58, *P* = 0.04) and a haplotype TGA (OR = 0.40, *P* = 0.01) were associated with reduced HCC risk after adjusting for lipid levels. In the immunohistochemistry experiment, we found that the protein expression of SOAT1 was significantly increased in the tumor compared with adjacent tissue (*P* < 0.001).

**Conclusion:**

This study revealed for the first time *SOAT1* genetic variation that associates with host susceptibility to HCC occurrence. Our results suggest a role of SOAT1 in the HCC development, which warrants further elucidation.

**Supplementary Information:**

The online version contains supplementary material available at 10.1186/s12885-021-08245-1.

## Background

Liver cancer is the second major cause of mortality for all types of cancer worldwide. Hepatocellular carcinoma (HCC) represents the largest proportion in liver cancer [1]. HCC incidence rates vary globally, with the majority of HCC cases occurring in East Asia and sub-Saharan Africa, due to the high prevalence of Hepatitis B virus (HBV) and hepatitis C virus (HCV). The United States and Northern Europe have a low HCC incidence but it has been increasing in recent years [2]. The major risk factors of HCC include chronic HBV and HCV infection, aflatoxin exposure, alcohol abuse, and non-alcoholic fatty liver disease (NAFLD). Among these factors, higher NAFLD prevalence is considered one of the key factors related to the increasing incidence of HCC in the low incidence areas and is expected to become the major cause of HCC in the future [3, 4].

Sterol O-acyltransferase (SOAT), also known as acyl-CoA: cholesterol acyltransferase (ACAT), is located in the endoplasmic reticulum membrane where it catalyzes cholesterol into cholesterol esters and plays an essential role in cholesterol homeostasis and bile acid biosynthesis [5, 6]. SOAT-mediated esterification of cholesterol prevents the toxic accumulation of free cholesterol in cell membrane [7]. SOAT1 is ubiquitously expressed in all tissues except the intestine. SOAT1 is the major enzyme with higher expression level and plays an important role in cholesterol homeostasis [5, 8–10]. A recent proteomic study performed in early-stage HBV-HCC patients revealed that SOAT1 plays an important role in a severe subtype of HCC [11]. They reported that HCC patients with more aggressive tumors and poorer prognosis had disrupted cholesterol metabolism and higher SOAT1 expression [11].

SOAT1 in HCC has been considered as a new promising target for HCC diagnosis and treatment [12, 13]. SOAT1 protein expression in HCC cell lines and inhibition of patient-derived tumor xenograft models demonstrated that SOAT1 suppression may be an effective HCC treatment [11]. Single nucleotide polymorphisms (SNPs) of *SOAT1* have been associated with cholesterol metabolism [14, 15]. However, the association between *SOAT1* SNPs and HCC has not been explored. Therefore, to assess whether *SOAT1* is related to risk of HCC occurrence, we explored the association of *SOAT1* gene missense variants with HCC susceptibility in a case-control design of biopsy proven HCC patients and healthy controls. To our knowledge, this is the first study to report a relationship between *SOAT1* genetic variants and HCC.

## Methods

### Study subject

The study included 221 cases diagnosed with HCC and 229 healthy control individuals from First Affiliated Hospital of Wenzhou Medical University between January 2010 and March 2019. There were 160 HBV infected HCC patients (72.4%) among all HCC cases. The self-reported ethnicity of participants was Han Chinese. All cases were confirmed by histopathology to have HCC. Inclusion criteria for healthy controls was no evidence of current hepatitis virus infection, no history of liver or other metabolic diseases, and no other malignancies. We obtained demographic and clinical data from review of medical charts.

The study was conducted in accordance with the Declaration of Helsinki. The Ethics Committee of Wenzhou Medical University approved this study. Informed consents were obtained from individuals of healthy controls. An IRB exemption was obtained from the National Institutes of Health Office of Human Subjects Research (OHSRP Review #12836) for using archived pathological specimens and the de-identified health information.

### Samples

We obtained all samples from the First Affiliated Hospital of Wenzhou Medical University. Achieved formalin-fixed and paraffin-embedded (FFPE) tissue from HCC patients were obtained from the Pathology Department and DNA was extracted from using the phenol extract method [16, 17]. Tumor grading and staging were classified by Barcelona.

Clinic liver Cancer Staging system (BCLC) [18]. We used the Universal Genomic DNA Extraction Kit Ver3.0 (Takara Bio, Japan) to extract genomic DNA from peripheral whole blood of healthy individuals.

### SNP selection

**Fig. 1:**
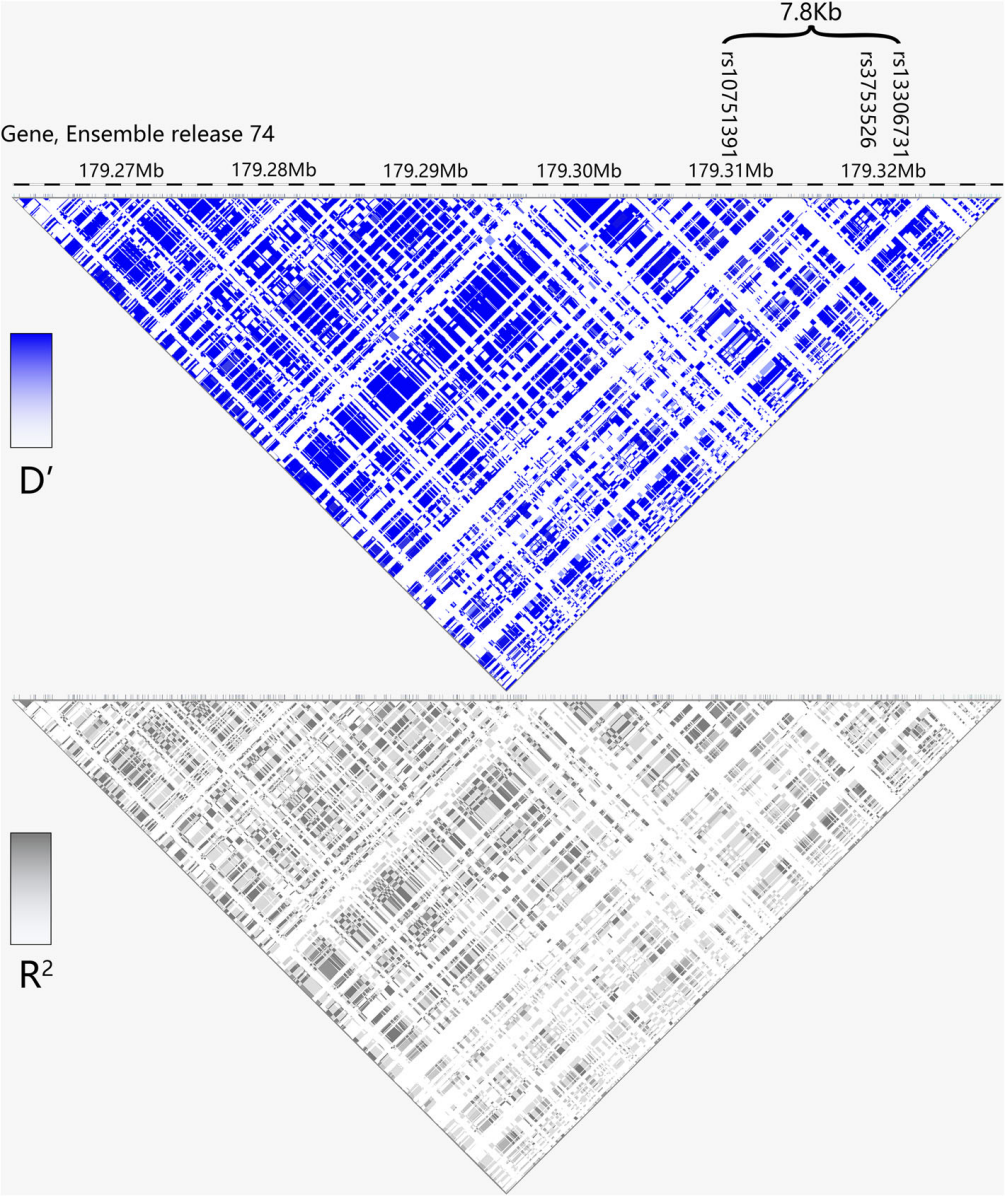
Linkage disequilibrium of SNPs in the *SOAT1* gene and locations of three SNPs tested in this study. The total length of the *SOAT1* gene is 65Kb, with three SNPs in this study covering a 7.8Kb region. The figure shows the degree of linkage (D′) and correlation (*r*^*2*^) of SNPs in the gene region of CHB. Most SNPs of *SOAT1* gene are in strong linkage disequilibrium (D′: 0.934 ± 0.156)

We selected variant sites from NCBI dbSNP and 1000Genomes database, based on the following criteria: (i) haplotype tagging SNPs; (ii) SNPs in the exonic regions of *SOAT1*; (iii) minor allele frequency (MAF) > 0.02 in Han Chinese from Beijing (CHB) in the 1000Genomes project database. Data from 1000Genomes indicated that most common variants in *SOAT1* were in strong linkage disequilibrium in three typical populations from China, Europe and Africa (Fig. S1). Then, we selected rs10753191 (synonymous amino acid change V323V), rs3753526 (synonymous amino acid change L475L) and rs13306731(nonsynonymous change Q526R (Gln526Arg)) from the 65 kb *SOAT1* gene, which covers a haplotype block approximately 7.8 kb from exon10 to exon16 (Fig. 1, Table 2). We analyzed the linkage disequilibrium of all 385 *SOAT1* variants in the CHB population available in the 1000Genomes project database. These SNPs are in strong linkage disequilibrium (Average D′: 0.934 ± 0.156 (mean ± sd), Fig. 1). We included all exonic SNPs in the *SOAT1* gene with MAF > 0.02 in CHB except one SNP rs11576517 (P199P) which was in high LD with rs10753191 (D′ = 0.84, *r*^*2*^ = 0.42). rs7547733 (F258F), which is common in the European population (MAF = 0.20) is absent in east Asians (including CHB).

### Genotyping

We conducted SNPs genotyping with TaqMan SNP Genotyping Assays using a real-time quantitative PCR method on a StepOnePlus Real-Time PCR System (Applied Biosystems, Foster City, California, USA) following Applied Biosystems protocols. Genotype call were made using the TaqMan Genotyper Software (Applied Biosystems). All the reactions were carried out in a total volume of 10ul containing TaqPath ProAmp Master Mix, SNP genotyping assay (20x), DNA-free water and genomic DNA. The PCR parameters were set as follows: 60 °C for 60s; 95 °C for 30s; 40 cycles at 95 °C for 15 s and 60 °C for 60s; 60 °C for 30s. About 10% random samples were duplicated for genotyping and the results were 100% concordant.

### Immunohistochemistry (IHC) for SOAT1 protein expression

We processed FFPE tissue samples to 3.5 μm to detect the expression of SOAT1 by IHC. Then we deparaffinized, rehydrated, and exposed those sections to 3% H_2_O_2_ solution for 10 min and performed antigen retrieval in the citrate buffer, followed by incubation with anti-SOAT1 (ABN66, Sigma) at 1:500 dilution at 4 °C overnight. Anti-rabbit secondary antibody was then applied to sections for 25 min at 37 °C. After that we conducted visualization with Diaminobenzidine (ZSGB-Bio). The intensity of IHC results was defined as negative (0), low (1), medium (2) and high (3), while the percentage of stained cells were defined as none (0), 1–25% (1), 25–50% (2) 50–75% (3) and > 75% (4). According to the product of intensity and percentage, we classified samples as low expression (0–6) and high expression (8–12).

### Statistical analysis

All statistical analyses were performed with R language [20] using RStudio Version 1.2.1335. We performed linkage disequilibrium (LD) and haplotype analysis with LD heatmap package [21] and Haplo.stats package [22]. The genotype distribution of all SNPs among control samples were conformed to Hardy-Weinberg equilibrium. Baseline characteristics of study subjects was d escribed as mean ± standard deviation (SD) or percentages. Significance of different groups was calculated with Fisher’s exact test or logistic regression. We conducted log transformation to non-normal distribution data before Wilcoxon rank sum test and logistic regression. Finally, we applied Fisher’s exact test and Wilcoxon-signed rank sum test to IHC scores. Results were considered significant for *P* value less than 0.05 and all tests were two-tailed.

#### HCC data from TCGA

Accessible transcriptomic data of 364 HCC patients with overall survival (OS) data from the TCGA were analyzed. Differences in overall survival (OS) were tested by Cox proportional hazards regression for the high or low, as divided by median, of SOAT1 mRNA levels measured by RNA-seq. Kaplan–Meier survival plots with hazard rates (HR) and log-rank *p*-values were calculated and plotted, separately for the White and Asian ethnic groups and also for the all ethnic groups (Plus black, *n* = 17), as implemented in K-M plotter [23].

## Results

### Characteristic of study subjects

The characteristics of the HCC cases (*n* = 221) and healthy controls (*n* = 229) are presented in Table 1. Age, sex or BMI distribution were similar between cases and controls (*P* > 0.05, Table 1). In the lipid profiles comparison, we observed that low density lipoprotein (LDL), high density lipoprotein (HDL), total cholesterol (TC) and triglyceride (TG) were all lower in HCC cases than healthy controls (*P* < 0.001, Table 1).

**Table 1:** Characteristics of HCC cases and controls

	Case (***n =*** 221)	Control (***n*** = 229)	***P*** value
Age, years^a^	57.29 ± 10.88	55.59 ± 11.64	0.109
Sex^b^	181 (81.90%)	170 (74.24%)	0.065
BMI^a^	23.04 ± 2.97	23.07 ± 2.25	0.936
TC (mmol/dl)^a^	3.82 ± 1.20	4.92 ± 0.85	< 0.001
TG (mmol/dl)^a^	1.12 ± 0.77	1.44 ± 0.85	< 0.001
LDL (mmol/dl)^a^	2.32 ± 0.90	2.77 ± 0.73	< 0.001
HDL (mmol/dl)^a^	0.97 ± 0.36	1.43 ± 0.36	< 0.001

^a^These data are described in the form of mean standard ± deviation and compared with t test

^b^percentage and *Chi*^2^ test

### Association of *SOAT1* SNPs with the risk of HCC

The primary information of three *SOAT1* SNPs (rs10753191, rs3753526, rs13306731) is displayed in Table 2. MAFs of SNPs in our controls were similar to MAFs in Han Chinese individuals (CHB population) from the 1000Genomes project [19].

**Table 2:** Characteristics of *SOAT1* SNPs and haplotypes

**SNP**	**rs10753191**	**rs3753526**	**rs13306731**	
Position^a^	179,312,752	179,319,541	179,320,578	
REF	C	C	A	
ALT	T	G	G	
Amino acid change	V323V	L475L	Q526R	
TF binding^b^	RFX5	No	No	
MAF^c^ in CHB^d^	0.403	0.408	0.306	
MAF in CEU^d^	0.091	0.040	0.040	
MAF in YRI^d^	0.361	0.116	0	
MAF in the control group	0.394	0.394	0.316	
Hardy-Weinberg Equilibrium *P* value in the control group	0.197	0.197	0.790	
**Haplotype**	**This Study**	**CHB**	**CEU**	**YRI**
CCA	0.628	0.592	0.909	0.639
TGG	0.303	0.306	0.040	0.000
TGA	0.069	0.097	0.000	0.116
CGA	0.000	0.005	0.000	0.000
TCA	0.000	0.000	0.051	0.245

^a^NC_000001.10 (chr1), Feb 2009 GRCh37/hg19)

^b^Transcriptional factor (TF) binding

^c^MAF: Minor allele frequency

^d^Genotype data of CHB, CEU, YRI are all come from 1000Genomes [19]

*CHB* individuals from unrelated Han Chinese individuals from Beijing, China, *CEU* residents of Utah with western European ancestry, *YRI* individuals from Yoruba population of African origin

The association of SNPs with HCC risk was presented in Table 3. Genotyping results showed rs10753191 and rs3753526 are in near absolute positive LD and therefore rs10753191 is a proxy for rs3753526. We evaluated the associations of the SNPs with HCC status using dominant, recessive, additive genetic models. We observed no significant associations in the minimally adjusted model adjusting for age and sex; however, after adjusting for lipid levels, carriers of rs10753191 T had a lower risk for HCC (OR = 0.583, 95% Confidence Interval (CI) 0.348–0.977, *P* = 0.041, dominant model).

**Table 3:** Associations of *SOAT1* SNPs with risk of HBV-HCC

	Case (***n =*** 221)	Control (***n =*** 229)	***P*** value	OR(95% CI)	***P*** value^**†**^	OR(95% CI)^**†**^	***P*** value^**‡**^	OR(95% CI)^**‡**^
rs10753191								
CC	94 (42.5%)	81 (35.4%)	Ref.	/	Ref.	/	Ref.	/
CT	97 (43.9%)	118 (51.5%)	0.103	0.708 (0.475–1.057)	0.107	0.716 (0.477–1.075)	0.035	0.564 (0.331–0.960)
TT	30 (13.6%)	30 (13.1%)	0.655	0.862 (0.479–1.550)	0.547	0.833 (0.460–1.508)	0.483	0.747 (0.331–1.689)
Dominant	/	/	0.123	0.739 (0.506–1.081)	0.123	0.739 (0.504–1.085)	**0.040**	0.583 (0.508–2.177)
Recessive	/	/	0.891	1.042 (0.605–1.794)	0.965	1.012 (0.586–1.749)	0.893	1.051 (0.508–2.177)
Additive	/	/	0.237	0.840 (0.634–1.122)	0.277	0.858 (0.651–1.131)	0.156	0.764 (0.526–1.108)
rs13306731								
AA	107 (48.4%)	106 (46.3%)	Ref.	/	Ref.	/	Ref.	/
AG	95 (43.0%)	106 (46.3%)	0.557	0.888 (0.604–1.306)	0.626	0.908 (0.614–1.341)	0.496	0.837 (0.502–1.397)
GG	19 (8.6%)	17 (7.4%)	0.858	1.107 (0.546–2.246)	0.865	1.064 (0.519–2.185)	0.687	1.207 (0.483–3.020)
Dominant	/	/	0.706	0.918 (0.634–1.330)	0.714	0.932 (0.642–1.355)	0.613	0.881 (0.538–1.442)
Recessive	/	/	0.729	1.173 (0.593–2.320)	0.706	1.141 (0.574–2.268)	0.558	1.299 (0.541–3.122)
Additive	/	/	0.750	0.963 (0.513–1.563)	0.898	0.981 (0.731–1.317)	0.892	0.974 (0.663–1.430)

^†^Adjusted by age, gender

^‡^Adjusted by age, gender, HDL, LDL, TC and TG

Dominant model: CC vs. CT + TT for rs10753191; AA vs. AG + GG for rs13306731

Recessive model: TT vs. CC + CT for rs10753191; GG vs. AA+AG for rs13306731

Additive model: CC vs. CT vs. TT for rs10753191; GG vs. AA vs. AG vs. GG for rs13306731

### Association of *SOAT1* haplotype consisting of three SNPs with the risk of HCC

All SNP data of patients and healthy controls was combined to determine the extent of linkage disequilibrium for the three SNPs (Additional file 1 Table S1). All three SNPs were in strong linkage disequilibrium (D′ = 1, *r*^*2*^ > 0.7). Additionally, we validated this result with other three typical populations from 1000 Genomes [19] (CHB, CEU and YRI) and all three SNPs were assigned to the same haplotype block (Additional file 1 Fig. S1).

Next, we used Haplo.stats [22] to obtain the haplotype assignments of samples. The association of haplotypes with HCC risk is presented in Table 4. None of the three haplotypes was associated with HCC susceptibility in the minimally adjusted model, but after adding lipids to the model, haplotype TGA was associated with decreased risk of HCC (OR = 0.395, 95%CI = 0.191–0.817, *P* = 0.012) (Table 4).

**Table 4:** Associations of the *SOAT1* haplotypes with the risk of HCC

	Case	Control	***P*** value	OR(95% CI)	***P*** value^**†**^	OR(95% CI)^**†**^	***P*** value^**‡**^	OR(95% CI)^**‡**^
CCA	285	280	Ref.	/	Ref.	/	Ref.	/
TGA	24	38	0.083	0.620 (0.363–1.062)	0.056	0.587 (0.340–1.013)	**0.012**	0.395 (0.191–0.817)
TGG	133	140	0.659	0.933 (0.699–1.246)	0.647	0.934 (0.698–1.250)	0.585	0.900 (0.618–1.312)

^†^Model 1, adjusted for age and sex

^‡^Model 2, model 1 with additional adjustment of HDL, LDL, TC and TG

### Association of *SOAT1* SNPs and haplotype with HCC characteristics (Table 5)

We compared characteristics of HCC patients (including LDL, HDL, TC, TG, tumor size, alpha-fetoprotein level (AFP), HBV status and pathological stage) between different *SOAT1* genotypes and haplotypes. There were no significant differences in lipid levels, triglycerides, tumor size, stage, or HBV status by genotype or haplotype. Carriers of the variant alleles for rs10753191 and rs13306731 were more likely to have elevated AFP levels (*P* = 0.014 and *P* = 0.010, respectively). Moreover, haplotype TGG was associated with a tendency of lower AFP level in the minimally adjusted model (*P* = 0.042), but when lipids were added to the model the association was attenuated (*P* = 0.065, Table 5). We did not find significant association of the *SOAT1* SNPs and haplotypes with lipid levels in HCC patients or healthy controls (*P* > 0.05, Additional file 1 Table S2).

**Table 5:** Association of the *SOAT1* SNPs with the characteristic of HCC patients, dominant model

	Reference genotype		Variant genotypes		***P*** value^**†**^		***P*** value^**‡**^
rs10753191/ rs3753526	CC/CC (*n* = 94)		CT&TT/CG&GG (*n* = 127)				
LDL (mmol/dl)	2.276 ± 0.879		2.353 ± 0.911		0.608		0.606
HDL (mmol/dl)	0.945 ± 0.391		0.984 ± 0.328		0.248		0.237
TC (mmol/dl)	3.803 ± 1.216		3.824 ± 1.190		0.668		0.664
TG (mmol/dl)	1.235 ± 0.952		1.037 ± 0.590		0.384		0.384
Size (cm)	3.987 ± 2.536		4.234 ± 2.892		0.533		0.534
AFP > 20 ng/ml	30 (31.9%)		56 (44.1%)		**0.009** ^§^		**0.014** ^¶^
HBsAg+	71 (75.5%)		89 (70.1%)		0.355^§^		0.228^¶^
Stage(0-B) ^&^	72 (76.6%)		87 (68.5%)		0.0.190^§^		0.146^¶^
rs13306731	AA (*n* = 107)		AG&GG (*n* = 114)				
LDL (mmol/dl)	2.262 ± 0.846		2.376 ± 0.943		0.993		0.993
HDL (mmol/dl)	0.937 ± 0.382		0.995 ± 0.328		0.230		0.219
TC (mmol/dl)	3.749 ± 1.176		3.880 ± 1.221		0.921		0.920
TG (mmol/dl)	1.201 ± 0.913		1.046 ± 0.597		0.246		0.246
size (cm)	3.999 ± 2.514		4.253 ± 2.951		0.518		0.520
AFP > 20 ng/ml	34 (31.8%)		52 (45.6%)		**0.003**^**§**^		**0.010** ^¶^
HBsAg+	79 (73.8%)		81 (71.1%)		0.498^§^		0.352^¶^
Stage(0-B)^&^	80 (74.8%)		79 (69.3%)		0.354^§^		0.311^¶^
Haplotype	CCA (*n* = 285)	TGG (*n* = 133)	TGA (*n* = 24)	TGG *P* value^†^	TGG *P* value^‡^	TGA *P* value^‡^	TGA *P* value^‡^
LDL (mmol/dl)	2.311 ± 0.911	2.349 ± 0.905	2.259 ± 0.640	0.702	0.774	0.785	0.863
HDL (mmol/dl)	0.955 ± 0.375	1.003 ± 0.318	0.912 ± 0.300	0.206	0.391	0.597	0.592
TC (mmol/dl)	3.829 ± 1.215	3.862 ± 1.221	3.393 ± 0.688	0.802	0.927	0.091	0.100
TG (mmol/dl)	1.178 ± 0.854	1.046 ± 0.587	0.870 ± 0.467	0.115	0.149	0.089	0.067
size (cm)	4.131 ± 2.715	4.228 ± 2.886	3.500 ± 2.077	0.755	0.808	0.311	0.182
AFP > 20 ng/ml	60 (21.1%)	38 (28.6%)	4 (16.7%)	**0.042**^§^	0.065^¶^	0.487^§^	0.555^¶^
HBsAg+	206 (72.3%)	95 (71.4%)	17 (70.8%)	0.857^§^	0.908^¶^	0.879^§^	0.634^¶^
Stage(0-B)^&^	202 (70.9%)	92 (69.2%)	7 (29.2%)	0.562^§^	0.593^¶^	0.642^§^	0.409^¶^

^†^One-way ANOVA

^‡^ANOVA adjusted for age and sex

^§^Logistic regression

^¶^Logistic regression adjusted for age and sex

^&^Stage 0-B versus C-D

### Association of SNPs with SOAT1 protein expression in HCC tumor and liver tissue

**Fig. 2:**
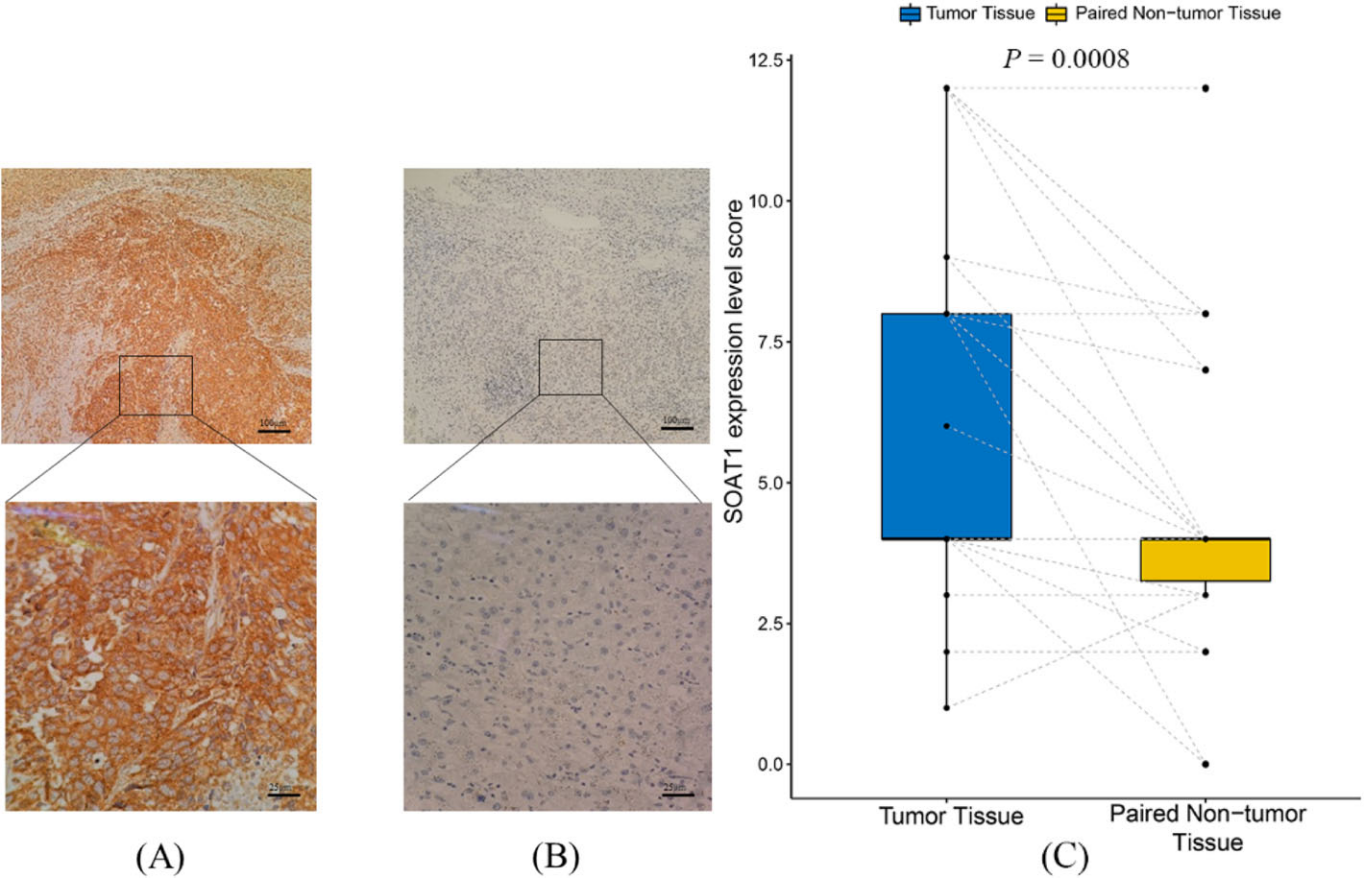
SOAT1 protein expression in HCC tumor and non-tumor tissues. **a** Presentative tumor immunohistochemistry staining of SOAT1 protein; **b** Presentative non-tumor immunohistochemistry staining of SOAT1 protein; **c** IHC score differences between tumor and non-tumor tissues (*P* < 0.001, paired Wilcoxon singed-rank test). Tumor and non-tumor Samples from the same patient are connected with dotted gray lines. The thickness of the dotted line represents different sample sizes

We measured the SOAT1 protein expression by immunohistochemistry (IHC) in HCC liver tissue samples and paired non-tumor tissue samples from 42 patients. Representative staining results are shown in Fig. 2. Immunoreactivity were mainly seen on the membrane plasma of tumor cells. A small number of lymphocytes were also weakly stained. We compared the differences in expression between hepatocytes and hepatocarcinoma cells. We conducted paired Wilcoxon signed-rank test for IHC score differences and found SOAT1 has a markedly higher expression level in tumor tissues compared to paired non-tumor tissues (*P* < 0.001, paired Wilcoxon singed-rank test, Fig. 2). We found no significant association of the SOAT1 protein expression by genotype, haplotype or pathological stages (Table 6).

**Table 6:** Association of SOAT1 expression with the characteristic of HCC patients

	Low expression	High expression	***P*** value^**†**^	OR	CI (95%)
**rs10753191**					
CC	8	9	Ref.	1	
CT	8	9	1	1	0.260–3.845
TT	3	5	1	1.481	0.265–8.267
Dominant model			1	1.131	0.328–3.898
Recessive model			0.709	0.675	0.139–3.283
**rs13306731**					
AA	9	9	Ref.	1	
AG	7	11	1	1	0.158–6346
GG	3	3	0.738	1.571	0.418–5.903
Dominant model			0.756	0.714	0.209–2.443
Recessive model			1	0.800	0.142–4.513
**Haplotype**					
CCA	24 (63.16%)	27 (58.70%)	Ref.	/	
TGA	1 (2.63%)	2 (4.35%)	1	1.778	0.151–20.863
TGG	13 (34.21%)	17 (36.96%)	0.819	1.162	0.469–2.881
**Stage**					
0-B	12	15	Ref.	/	
C-D	7	8	1	1.094	0.308–3.883

^†^Fisher exact test

### Functionality of the *SOAT1* SNPs

SNPs rs10753191 (V323V) and rs3753526 (L475L) do not change amino acid, and Q526R (rs13306731) is a predicted benign amino acid change based on Polyphen-2 program, (http://genetics.bwh.harvard.edu/pph2/index.shtml). We used the Position Weight Matrix model (PMW) based SNP2TFBS (https://ccg.epfl.ch/snp2tfbs/) in-silico bioinformatic tool to assess SNP impact on transcription factor binding (TFB) [24, 25]. While rs3753526 and rs13306731 have no effect on TFB, rs10753191 was predicted to significantly enhance binding to DNA-binding protein regulatory factor X-5 (RFX5). RFX5 has been reported to significantly upregulated in HCC tumors and cell lines [26]. Thus, rs10753191 may affect TFB and possibly gene regulation.

#### HCC data from TCGA

**Fig. 3:**
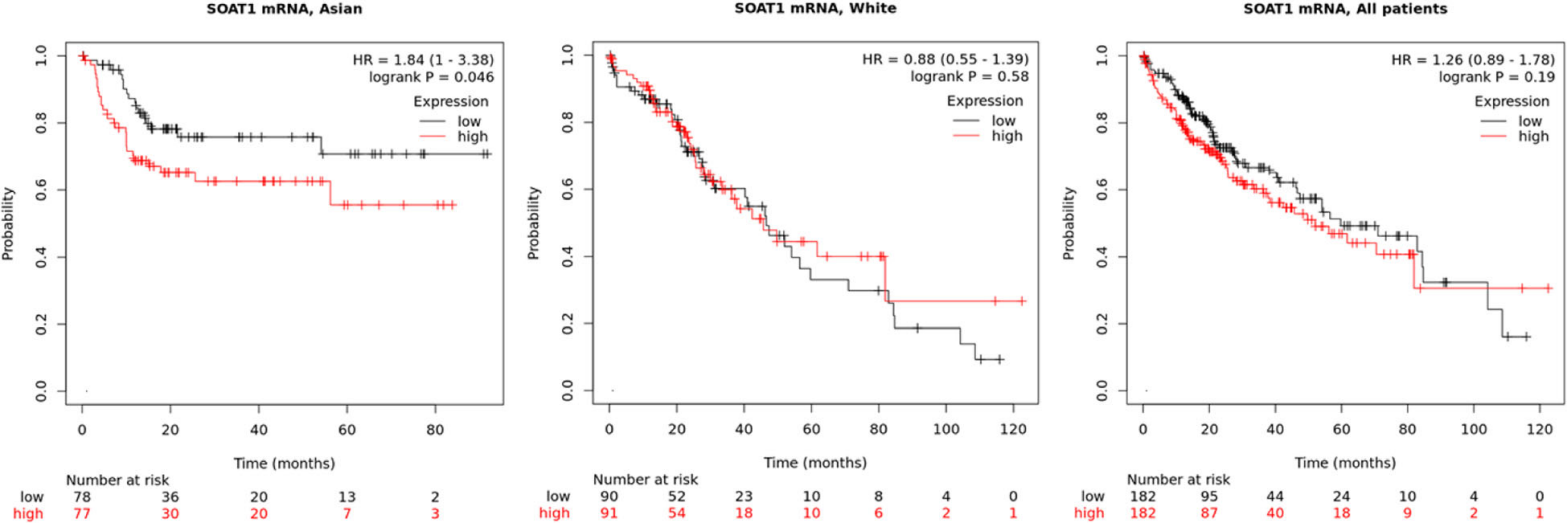
Impact of *SOAT1* mRNA expression level on survival of HCC patients in TCGA, stratified by ethnic group. **a**. Asian; **b**. White; **c**. In all patients. HR, hazard rate

A high level of *SOAT1* mRNA expression level was associated with a marginal significantly shorter overall survival in Asians (*P* = 0.046). However, there was not association in White/Caucasians (*P* = 0.58), or in all ethnic groups combined (Asian, White and black, *P* = 0.17, Fig. 3). There was no significant impact of *SOAT1* expression on OS when restricting the survival analysis to those HBV-infected (*n* = 150, *P* = 0.32).

## Discussion

In this study we found a protective association between two variant *SOAT1* alleles and a haplotype carrying these alleles and HCC after adjusting for lipid levels. We also observed a markedly higher protein expression level of SOAT1in tumor tissues compared to paired non-tumor tissues. A high SOAT1 mRNA expression level was further revealed to be associated with a shorter overall survival of HCC patients from the TCGA data in Asians but not in Caucasians, suggesting a population-specific role of SOAT1 in HCC. The ethnic/population specific role of SOAT1 is worthy particular attention as it is considered as a promising drug target of HCC [11]. SOAT1 is the key protein in catalyzing the formation of fatty acid-cholesterol esters [5] and we observed lower lipid levels in HCC cases compared to healthy controls. Previous studies suggest that cholesterol metabolism plays an important role in the progression of HCC [27–31]. A proteomics study found that HCC patients with disrupted cholesterol metabolism and high expression of SOAT1 tend to have a poorer prognosis [11]. The same study found that in a patient-derived tumor xenograft mouse model suppression of SOAT1 reduced tumor size [11]. Thus, SOAT1 may be a new target for HCC treatment. Our results suggest that SOAT1 may influence HCC risk through regulation of lipid metabolism.

We observed that LDL, HDL, TC and TG levels were lower in HCC cases than in normal controls. This finding is agreement with other studies which show that lipids and triglyceride levels are decreased in patients with HCC [27]. The relationship between lipid and HCC is complex. On the one hand, lipid metabolism alteration can be a consequence of HCC development. Cancer cachexia is frequently observed in cancer patients and characterizes by reduction in fat stores, elevated carbohydrate utilization and protein degradation. High growth rate of cancer cells leads to hypoxia and increased energy demand, and eventually promotes fatty-acid oxidation which will deplete fat storage [32–34]. On the other hand, dysregulated lipid metabolism may promote HCC, due to impaired insulin and IGF-1 pro-tumorigenic growth factors [35, 36].

Several *SOAT1* SNPs are associated with cholesterol metabolism [14, 15]. A meta-analysis by Andrew et al. found rs4421551 is associated with HDL level [14]. Wu et al. reported that carriers of rs1044925 variant genotypes had lower serum TC, LDL and ApoB levels than the reference genotype [15]. Both of these SNPs are in linkage disequilibrium with the three SNPs in this study (D′ = 1). It remains possible that the variant allele at rs10753191 or its proxy rs3753526 or other variants in LD with these SNPs may reduce the risk of HCC through lowering the lipid levels.

In an analysis of association between genotype and HCC related phenotypes, a lower level of AFP in serum decrease tended to associate with the SNPs employed in this study. Due to the low sensitivity and specificity of AFP for HCC diagnosis, this association may have utility in risk assessment.

Jiang et al. reported that a high SOAT1 expression increases the severity of HCC patients [11]. Our immunohistochemistry results also found higher expression of SOAT1 in tumor tissue compared to paired non-tumor tissue. However, our analysis showed no significant association between SOAT1 protein expression by genotypes, haplotypes or BCLC stages (Table 6).

Our results suggest *SOAT1* variants may modestly modify HCC risk, possibly through the lipid metabolism pathway. On the other hand, our results also suggest that the impact of SOAT1 on HCC might be limited, calling for continuing search of other HCC host proteins involved in this multigenic heterogenous cancer.

Several limitations in this case-control study should be noted. HCC development is a complex process linked to multiple factors including age, sex, alcohol consumption, environment toxins, HBV and HCV viral levels, and diet. This study did not adjust for all of these confounding factors. Second, our sample size was not large enough to detect small effect sizes. Our sample size had adequate power of 80% to detect genotype relative risk of 1.88 for SNP rs10753191 (MAF 0.31, dominant model) or 2.05 for rs10753191(MAF 0.39) based on calculation using Genetic Association Study (GAS) Power Calculator [37]. Third, this study employed multiple genetic models and explanatory variables which could cause inflation of type 1 errors. The prior biological evidence of the gene-disease relationship is in favor of presence of a weak genetic association. Fourth, we only queried 2 independent SNPs and three haplotypes so it is quite likely that our SNPs and haplotypes are tracking through LD with other variants that may be functional. These SNPs may not be causal or functional by themselves. In addition, the frequencies of variants and haplotype structure of *SOAT1* vary among populations, thus their effects in other populations may also vary. Finally, we did not have a replication cohort to validate our results; therefore, further studies are warranted to validate our results and to identify putative causal variants through fine mapping and functional studies. Potential relationship of HBV infection interacting with SOAT1 to contribute to HCC tumorigenesis is also an important topic for the future research.

## Conclusion

In conclusion, this study is the first to implicate *SOAT1* genetic variation that modifies HCC susceptibility. Studies with larger sample size, stratified by confounding factors and protein levels of SOAT1, should be conducted to validate its role in developing of HCC.

## Supplementary Information

**Additional file 1.**

## Data Availability

The datasets used and/or analyzed during the current study are available from the corresponding author on reasonable request.

## Abbreviations

HBV: Hepatitis B virus
HCC: Hepatocellular carcinoma
SOAT: Sterol O-acyltransferase
SNP: Single Nucleotide Polymorphism
NAFLD: Non-alcoholic fatty liver disease
FFPE: Formalin-fixed and paraffin-embedded
BCLC: Barcelona Clinic Liver Cancer
MAF: Minor allele frequency
LD: Linkage disequilibrium
LDL: Low density lipoprotein
HDL: High density lipoprotein
TC: Total cholesterol
TG: Triglyceride
AFP: Alpha-fetoprotein
HBsAg: Hepatitis B s antigen
