# Supplementary material for: Impacts of the SOAT1 genetic variants and protein expression on HBV-related hepatocellular carcinoma
Source: BMC Cancer. 2021 May 26;21:615. doi: 10.1186/s12885-021-08245-1 (PMC8152151; doi:10.1186/s12885-021-08245-1)
Supplement: Supplementary file 1 — Additional file 1. [file 12885_2021_8245_MOESM1_ESM.docx]

**Table S1.** **Linkage disequilibrium of the *SOAT1* SNPs**

|  | rs10753191/rs3753526 | | | rs13306731 |
| --- | --- | --- | --- | --- |
| Current Study | |  |  |  |
| rs10753191 | 1 (0.999) | | | 1 (0.734) |
| rs3753526 | NA | | | 1 (0.734) |
| rs13306731 | NA | | | NA |
| CHB |  | | |  |
| rs10753191 | 1 (0.979) | | | 1 (0.653) |
| rs3753526 | NA | | | 1 (0.640) |
| rs13306731 | NA | | | NA |
| CEU |  | | |  |
| rs10753191 | 0.998 (0.419) | | | 0.998 (0.419) |
| rs3753526 | NA | | | 0.998 (0.996) |
| rs13306731 | NA | | | NA |
| YRI |  | | |  |
| rs10753191 | 0.999 (0.231) | | | NA |
| rs3753526 | NA | | | NA |
| rs13306731 | NA | | | NA |

*Numbers in the table represent D’ value and numbers in brackets represent r^2^. R package Haplo.stats was used to process data. Genotype data of CHB, CEU, YRI are all come from 1000Genomes[21];

Table S2. Association of the *SOAT1* SNPs and haplotype with lipid levels

| **HCC** | | | | | | | |
| --- | --- | --- | --- | --- | --- | --- | --- |
| rs10753191/rs3753526 | | | | | | | |
|  | CC/CC  (n=84) | CT/CG  (n=97) | TT/GG  (n=30) | CT/CG  *P* value^†^ | CT/CG  *P* value^‡^ | TT/GG  *P* value^†^ | TT/GG  *P* value^‡^ |
| LDL | 2.276±0.879 | 2.381±0.974 | 2.258±0.664 | 0.443 | 0.447 | 0.922 | 0.902 |
| HDL | 0.945±0.391 | 0.975±0.346 | 1.014±0.262 | 0.581 | 0.865 | 0.381 | 0.577 |
| TC | 3.803±1.216 | 3.88±1.225 | 3.640±1.063 | 0.670 | 0.756 | 0.524 | 0.482 |
| TG | 1.235±0.952 | 1.066±0.616 | 0.939±0.490 | 0.154 | 0.177 | 0.117 | 0.147 |
| rs13306731 | | | | | | | |
|  | AA(n=107) | AG(n=95) | GG(n=19) | AG *P* value^†^ | AG *P* value^‡^ | GG *P* value^†^ | GG *P* value^‡^ |
| LDL | 2.262±0.846 | 2.413±0.992 | 2.189±0.633 | 0.729 | 0.750 | 0.845 | 0.847 |
| HDL | 0.937±0.382 | 0.984±0.342 | 1.052±0.250 | 0.850 | 0.903 | 0.599 | 0.666 |
| TC | 3.749±1.176 | 3.904±1.219 | 3.757±1.255 | 0.519 | 0.506 | 0.540 | 0.639 |
| TG | 1.201±0.913 | 1.047±0.611 | 1.042±0.537 | 0.105 | 0.129 | 0.444 | 0.382 |
| **Control** | | | | | | | |
| rs10753191/rs3753526 | | | | | | | |
|  | CC/CC  (n=81) | CT/CG  (n=118) | TT/GG  (n=30) | CT/CG  *P* value^†^ | CT/CG  *P* value^‡^ | TT/GG  *P* value^†^ | TT/GG  *P* value^‡^ |
| LDL | 2.808±0.732 | 2.731±0.726 | 2.802±0.718 | 0.463 | 0.385 | 0.969 | 0.965 |
| HDL | 1.440±0.320 | 1.401±0.372 | 1.516±0.381 | 0.435 | 0.326 | 0.296 | 0.192 |
| TC | 4.985±0.864 | 4.858±0.848 | 5.002±0.820 | 0.303 | 0.230 | 0.926 | 0.874 |
| TG | 1.433±0.798 | 1.445±0.872 | 1.416±0.939 | 0.926 | 0.898 | 0.924 | 0.800 |
| rs13306731 | | | | | | | |
|  | AA(n=106) | AG(n=106) | GG(n=17) | AG *P* value^†^ | AG *P* value^‡^ | GG *P* value^†^ | GG *P* value^‡^ |
| LDL | 2.837±0.735 | 2.691±0.743 | 2.816±0.492 | 0.768 | 0.69 | 0.856 | 0.859 |
| HDL | 1.422±0.334 | 1.426±0.38 | 1.502±0.353 | 0.386 | 0.279 | 0.283 | 0.092 |
| TC | 5.008±0.889 | 4.836±0.823 | 4.926±0.736 | 0.437 | 0.329 | 0.981 | 0.925 |
| TG | 1.464±0.793 | 1.439±0.921 | 1.258±0.783 | 0.757 | 0.793 | 0.481 | 0.353 |
| **Haplotype** | | | | | | | |
| **HCC** | |  |  |  |  |  |  |
|  | CCA(n=285) | TGG(n=133) | TGA(n=24) | TGG  *P* value^†^ | TGG  *P* value^‡^ | TGA  *P* value^†^ | TGA  *P* value^‡^ |
| LDL | 2.311±0.911 | 2.349±0.905 | 2.259±0.640 | 0.702 | 0.774 | 0.785 | 0.863 |
| HDL | 0.955±0.375 | 1.003±0.318 | 0.912±0.300 | 0.206 | 0.391 | 0.597 | 0.592 |
| TC | 3.829±1.215 | 3.862±1.221 | 3.393±0.688 | 0.802 | 0.927 | 0.091 | 0.100 |
| TG | 1.178±0.854 | 1.046±0.587 | 0.870±0.467 | 0.115 | 0.149 | 0.089 | 0.067 |
| **Control** | |  |  |  |  |  |  |
|  | CCA(n=280) | TGG(n=140) | TGA(n=38) | TGG  *P* value^†^ | TGG  *P* value^‡^ | TGA  *P* value^†^ | TGA  *P* value^‡^ |
| LDL | 2.776±0.728 | 2.721±0.690 | 2.879±0.819 | 0.463 | 0.476 | 0.418 | 0.443 |
| HDL | 1.424±0.342 | 1.445±0.372 | 1.420±0.397 | 0.563 | 0.510 | 0.957 | 0.912 |
| TC | 4.932±0.856 | 4.858±0.799 | 5.088±0.955 | 0.393 | 0.405 | 0.299 | 0.321 |
| TG | 1.438±0.827 | 1.395±0.887 | 1.583±0.893 | 0.623 | 0.559 | 0.316 | 0.324 |

^†^One-way ANOVA;

^‡^ANOVA adjusted for age and sex.


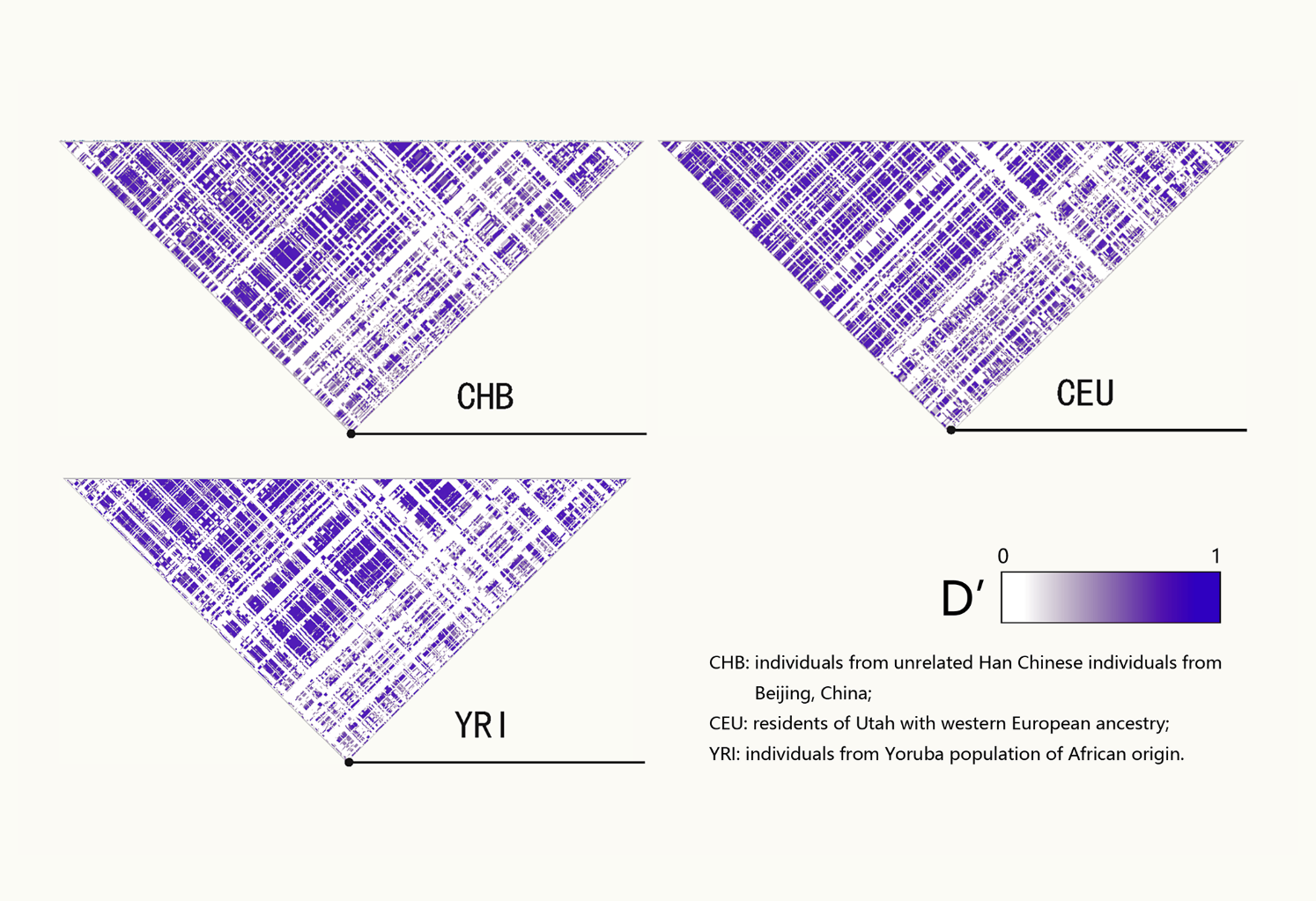


**Figure S1.** Linkage disequilibrium map of SNPs in the SOAT1 gene in different populations
